# Severe Pneumonia Caused by *Corynebacterium striatum* in Adults, Seoul, South Korea, 2014–2019

**DOI:** 10.3201/eid2811.220273

**Published:** 2022-11

**Authors:** Yun Woo Lee, Jin Won Huh, Sang-Bum Hong, Jiwon Jung, Min Jae Kim, Yong Pil Chong, Sung-Han Kim, Heungsup Sung, Kyung-Hyun Do, Sang-Oh Lee, Chae-Man Lim, Yang Soo Kim, Younsuck Koh, Sang-Ho Choi

**Affiliations:** University of Ulsan College of Medicine, Seoul, South Korea

**Keywords:** pneumonia, bacteria, respiratory infections, Corynebacterium striatum, intensive care units, incidence, mortality, South Korea

## Abstract

We investigated the proportion and characteristics of severe *Corynebacterium striatum* pneumonia in South Korea during 2014–2019. As part of an ongoing observational study of severe pneumonia among adult patients, we identified 27 severe *C. striatum* pneumonia cases. Most (70.4%) cases were hospital-acquired, and 51.9% of patients were immunocompromised. *C. striatum* cases among patients with severe hospital-acquired pneumonia (HAP) increased from 1.0% (2/200) during 2014–2015 to 5.4% (10/185) during 2018–2019, but methicillin-resistant *Staphylococcus aureus* (MRSA) infections among severe HAP cases decreased from 12.0% to 2.7% during the same timeframe. During 2018–2019, *C. striatum* was responsible for 13.3% of severe HAP cases from which bacterial pathogens were identified. The 90-day mortality rates were similarly high in the *C. striatum* and MRSA groups. *C. striatum* was a major cause of severe HAP and had high mortality rates. This pathogen is emerging as a possible cause for severe pneumonia, especially among immunocompromised patients.

## Abstract 2

Most (70.4%) cases were hospital-acquired, and 51.9% of patients were immunocompromised.

*Corynebacterium striatum* is a nonlipophilic, fermentative coryneform bacterium that commonly occupies the normal flora of the skin and oropharynx (*1*). Although *C. striatum* isolated from clinical specimens has frequently been considered a contaminant, it is increasingly recognized as a pathogen of various infections, including central line-associated bacteremia (*2*), endocarditis (*3*), and pleuropulmonary infection (*4*–*6*). In 1980, *C. striatum* was reported as a cause of pleuropulmonary infection in a patient with chronic lymphocytic leukemia (*4*). In 2018, a group of researchers in the United States reported 3 cases of community-acquired pneumonia (CAP) in which *Corynebacterium* species were the predominant isolate and suggested that *Corynebacterium* species are a noteworthy clinical cause of pneumonia (*6*). However, scarce information is available on the incidence, clinical characteristics, and outcomes of severe *C. striatum* pneumonia in critically ill adult patients, because previous studies included <5 patients with severe *C. striatum* pneumonia, except those reporting hospital outbreak events.

Methicillin-resistant *Staphylococcus aureus* (MRSA) is a major cause of severe hospital-acquired pneumonia (HAP), and the clinical characteristics and outcomes of severe MRSA pneumonia are well-documented. Therefore, comparing *C. striatum* and MRSA pneumonia could clarify the clinical characteristics of *C. striatum* pneumonia for clinicians. We investigated the proportion, clinical characteristics, and outcomes of severe *C. striatum* pneumonia in adults and compared those aspects with those for severe MRSA pneumonia.

## Methods

### Study Design, Setting, Data Collection, and Patient Selection

This study is part of an ongoing prospective observational study on severe pneumonia in critically ill adult (>16 years of age) patients at Asan Medical Center, a 2,700-bed tertiary referral center in Seoul, South Korea. Since March 2010, we have prospectively identified all adult patients admitted to the 28-bed medical intensive care unit (ICU) who were clinically suspected of having severe pneumonia and monitored them until hospital discharge (*7*–*10*). We collected data on patient demographics; underlying diseases or conditions; category of pneumonia; initial clinical manifestations; laboratory, microbiologic, and radiologic findings; treatment; complications; and mortality rates. For this study, we investigated patients with severe *C. striatum* pneumonia who were admitted to the medical ICU during January 2014–December 2019. This study was approved by the institutional review board of Asan Medical Center (IRB no. 2010–0079), which waived the need for informed consent due to the observational nature of the study.

### Definitions

We defined and categorized pneumonia as previously stated (*11*–*13*). We defined severe pneumonia as the necessity for mechanical ventilation or having septic shock at ICU admission (*12*). We defined sepsis and septic shock according to Sepsis-3 criteria (*14*). We defined immunocompromised state as described previously (*15*).

### *C. striatum* Identification and Antimicrobial Susceptibility Testing 

We cultured sputum specimens on a 5% sheep blood plate and MacConkey agar (Synergy Innovation, http://www.synergyinno.com). When coryneform gram-positive bacilli were isolated, we identified and performed antimicrobial susceptibility testing for specimens that were urea positive or from the ICU (*16*). We quantitatively cultured bronchoalveolar lavage specimens on chocolate agar and identified and performed susceptibility testing when coryneform gram-positive bacilli exclusively grew at >10^4^ CFU/mL (*16*). Until August 2015, our facility used the triple sugar iron, motility, API Coryne (bioMérieux-Vitek, https://www.biomerieux.com) system to identify coryneform gram-positive rods. In September 2015, our facility began using matrix-assisted laser desorption/ionization time-of-flight mass spectrometry (Bruker Daltonik, https://www.bruker.com). We determined antimicrobial susceptibility profiles by ETEST (bioMérieux-Vitek) with MHF medium (Mueller-Hinton agar with 5% horse blood + 20 mg/L β-NAD; bioMérieux-Vitek). We used the Clinical and Laboratory Standards Institute M45 guideline for interpreting susceptibility test results (*17*) and defined multidrug resistance as resistance to >3 antimicrobial drug families.

### Statistical Analysis

We compared patient demographics, underlying diseases and conditions, and clinical and laboratory parameters between the *C. striatum* group and the MRSA group. We used χ^2^ or Fisher exact test to compare categorical variables and Student *t*-test or Mann-Whitney U test to compare continuous variables. We analyzed changes in the proportions of pneumonic pathogens over time by using a χ^2^ test for trend. We performed all analyses in SPSS Statistics 24.0 (IBM Corp., https://www.ibm.com) and considered p<0.05 statistically significant.

## Results

### Demographics, Underlying Diseases and Conditions, and Pneumonia Categories

During the study period, we identified a total of 1,740 patients with severe pneumonia. Among them, 27 had severe *C. striatum* pneumonia and 103 had severe MRSA pneumonia (Table 1). The median patient age in the *C. striatum* group was 72.0 years and in the MRSA group was 71.0 years. Solid cancer, diabetes mellitus, and structural lung diseases were the most common underlying conditions in both groups. More patients in the *C. striatum* group were immunocompromised (51.9% vs. 26.2%; p = 0.01). Most (70.4%) patients in the *C. striatum* group had HAP, 14.8% had healthcare-associated pneumonia (HCAP), 11.1% had ventilator-associated pneumonia, and 3.7% had CAP. HAP was significantly more common in the *C. striatum* group than the MRSA group (70.4% vs. 42.7%; p = 0.01); HCAP was more common in the MRSA group (32.0% vs. 14.8%; p = 0.08), albeit without statistical significance.

**Table 1:** Characteristics of adult patients with severe pneumonia caused by *Corynebacterium striatum*, Seoul, South Korea, 2014–2019*

Characteristics	Total, n = 130	*C. striatum*, n = 27	MRSA, n = 103	p value
Sex				
M	92 (70.8)	18 (66.7)	74 (71.8)	0.60
F	38 (29.2)	9 (33.3)	33 (32.0)	
Median age (interquartile range)	71.0 (63.8–77.0)	72.0 (66.0–80.0)	71.0 (63.0–76.0)	0.17
Underlying disease or condition†				
Solid cancer	32 (24.6)	4 (14.8)	28 (27.2)	0.18
Diabetes mellitus	30 (23.1)	6 (22.2)	24 (23.3)	0.91
Structural lung disease	24 (18.5)	4 (14.8)	20 (19.4)	0.78
Chronic obstructive lung disease	12 (9.2)	3 (11.1)	9 (8.7)	0.71
Interstitial lung disease	5 (3.8)	0	5 (4.9)	0.58
Bronchiectasis	4 (3.1)	0	4 (3.9)	0.58
Destroyed lung due to tuberculosis	1 (0.8)	0	1 (1.0)	1.00
Pneumoconiosis	1 (0.8)	0	1 (1.0)	1.00
Bronchiolitis obliterans	1 (0.8)	1 (3.7)	0	0.21
Hematologic malignancy	13 (10.0)	5 (18.5)	8 (7.8)	0.14
Liver cirrhosis	11 (8.5)	2 (7.4)	9 (8.7)	1.00
End-stage renal disease	7 (5.4)	2 (7.4)	5 (4.9)	0.64
Chronic renal failure	6 (4.6)	3 (11.1)	3 (2.9)	0.10
Congestive heart failure	3 (2.3)	1 (3.7)	2 (1.9)	0.51
Alcoholism	2 (1.5)	0	2 (1.9)	1.00
Cerebrovascular attack	12 (9.2)	5 (18.5)	7 (6.8)	0.13
Solid organ transplantation	2 (1.5)	0	2 (1.9)	0.63
Hematopoietic stem cell transplantation	3 (2.3)	2 (7.4)	1 (1.0)	0.11
Immunocompromised state‡	41 (31.5)	14 (51.9)	27 (26.2)	0.01
Recent chemotherapy	23 (17.7)	7 (25.9)	16 (15.5)	0.26
Recent surgery, <1 mo	19 (14.6)	2 (7.4)	17 (16.5)	0.36
Active smoker	10 (7.7)	1 (3.7)	9 (8.7)	0.69
Neutropenia, <500 cells/mL	8 (6.2)	4 (14.8)	4 (3.9)	0.06
Category of pneumonia				
Community-acquired	6 (4.6)	1 (3.7)	5 (4.9)	1.00
Healthcare-associated	37 (28.5)	4 (14.8)	33 (32.0)	0.08
Hospital-acquired	63 (48.5)	19 (70.4)	44 (42.7)	0.01
Ventilator-associated	24 (18.5)	3 (11.1)	21 (20.4)	0.40

*Values are no. (%) except as indicated. MRSA, methicillin-resistant *Staphylococcus aureus*. †Patients could have >1 underlying disease or condition. ‡Defined as >1 of the following conditions: daily receipt of immunosuppressants, including corticosteroids; HIV infection; solid organ or hematopoietic stem cell transplant recipient; receipt of chemotherapy for underlying malignancy during the previous 6 months; or underlying immune deficiency disorder.

### Bacterial Pathogens Identified in Severe HAP Patients

We identified bacterial pathogens in 565 patients who had severe HAP during 2014–2019 (Table 2). The proportion of severe MRSA HAP decreased significantly, from 12.0% (24/200) in 2014–2015 to 2.7% (5/185) in 2018–2019 (p<0.01), whereas the proportion of severe *C. striatum* HAP increased significantly, from 1.0% (2/200) in 2014–2015 to 5.4% (10/185) in 2018–2019 (p<0.001). Among 75 HAP cases from which bacterial pathogens were identified in 2018–2019, *C. striatum* was responsible for 13.3% (10/75) of cases, which was the fourth most common pathogen, after *Acinetobacter baumannii* (30.7%), *Klebsiella pneumoniae* (21.3%), and *Pseudomonas aeruginosa* (14.7%).

**Table 2:** Bacterial pathogens detected among 565 adult patients with severe hospital-acquired pneumonia, Seoul, South Korea, 2014–2019

Pathogens identified	No. (%) patients				p value*
	2014–2015, n = 200	2016–2017, n = 180	2018–2019, n = 185	Total, n = 565	
Total	88 (44.0)	66 (36.7)	75 (40.5)	229 (40.5)	0.35
*Staphylococcus aureus*	27 (13.5)	15 (8.3)	8 (4.3)	50 (8.8)	<0.01
Methicillin-susceptible	3 (1.5)	0	3 (1.6)	6 (1.1)	0.24
Methicillin-resistant	24 (12.0)	15 (8.3)	5 (2.7)	44 (7.8)	<0.01
*Corynebacterium striatum*	2 (1.0)	7 (3.9)	10 (5.4)	19 (3.4)	0.05
*Streptococcus pneumoniae*	4 (2.0)	2 (1.1)	1 (0.5)	7 (1.2)	0.43
*Legionella pneumophila*	1 (0.5)	1 (0.6)	0	2 (0.4)	0.61
*Moraxella catarrhalis*	0	0	1 (0.5)	1 (0.2)	0.36
*Streptococcus pyogenes*	0	1 (0.6)	0	1 (0.2)	0.34
*Nocardia* species	0	0	1 (0.5)	1 (0.2)	0.36
Enteric gram-negative bacilli	18 (9.0)	22 (12.2)	20 (10.8)	60 (10.6)	0.59
* Klebsiella pneumoniae*	13 (6.5)	14 (7.8)	16 (8.6)	43 (7.6)	0.73
* Escherichia coli*	4 (2.0)	4 (2.2)	3 (1.6)	11 (1.9)	0.92
* Enterobacter cloacae*	1 (0.5)	3 (1.7)	2 (1.1)	6 (1.1)	0.54
* Citrobacter freundii*	1 (0.5)	2 (1.1)	0	3 (0.5)	0.34
* Klebsiella oxytoca*	0	0	2 (1.1)	2 (0.4)	0.13
* Hafnia alvei*	0	0	1 (0.5)	1 (0.2)	0.36
Nonenteric gram-negative bacilli	47 (23.5)	22 (12.2)	37 (20.0)	106 (18.8)	0.02
* Acinetobacter baumannii*	24 (12.0)	13 (7.2)	23 (12.4)	60 (10.6)	0.20
* Pseudomonas aeruginosa*	19 (9.5)	6 (3.3)	11 (5.9)	36 (6.4)	0.047
* Stenotrophomonas maltophilia*	4 (2.0)	2 (1.1)	7 (3.8)	13 (2.3)	0.22
* Burkholderia cepacia*	0	0	1 (0.5)	1 (0.2)	0.36
* Acinetobacter lwoffii*	0	1 (0.6)	0	1 (0.2)	0.34
* Chryseobacterium indologenes*	0	1 (0.6)	0	1 (0.2)	0.34
* Chryseobacterium meningosepticum*	1 (0.5)	0	0	1 (0.2)	0.40
* Chlamydia pneumoniae*	1 (0.5)	0	0	1 (0.2)	0.40

*p value based on χ^2^ test for trend.

### Coinfections

We identified co-infection pathogens in 13 (48.1%) patients in the *C. striatum* group and 37 (35.9%) patients in the MRSA group (p = 0.25) (Table 3). Co-infection with other bacteria was more common in the MRSA group (25.2% vs. 7.4%; p = 0.045), whereas viral co-infection was more common in the *C. striatum* group (33.3% vs. 14.6%; p = 0.047). Fungal co-infection, which included 4 *Aspergillus* species and 1 *Pneumocystis jirovecii*, was only found in the *C. striatum* group (14.8% vs. 0%; p<0.01).

**Table 3:** Additional pathogens detected among adult patients with severe *Corynebacterium striatum* pneumonia and methicillin-resistant *Staphylococcus aureus* pneumonia, Seoul, South Korea, 2014–2019*

Pathogens	No. (%) co-infecting pathogens			p value
	Total, n = 130	*C. striatum*, n = 27	MRSA, n = 103	
Any	50 (38.5)	13 (48.1)	37 (35.9)	0.25
Other bacteria	28 (21.5)	2 (7.4)	26 (25.2)†	0.045
* Pseudomonas aeruginosa*	7	0	7	
* Acinetobacter baumannii*	6	0	6	
* Klebsiella pneumoniae*	5	0	5	
* Escherichia coli*	4	1	3	
* Haemophilus influenzae*	2	0	2	
* Streptococcus pneumoniae*	2	0	2	
* Citrobacter freundii*	1	0	1	
* Enterobacter cloacae*	1	1	0	
* Elizabethkingia meningosepticum*	1	0	1	
* Klebsiella aerogenes*	1	0	1	
* Stenotrophomonas maltophilia*	1	0	1	
Virus	24 (18.5)	9 (33.3)‡	15 (14.6)§	0.047
Influenza virus	8	4	4	
Influenza virus A	3	3	0	
Influenza virus B	1	1	1	
Parainfluenza virus type 3	4	1	3	
Rhinovirus	3	1	2	
Adenovirus	3	1	2	
Respiratory syncytial virus	2	1	1	
Respiratory syncytial virus A	1	1	0	
Respiratory syncytial virus B	1	0	1	
Human coronavirus	2	1	1	
229E	1	1	0	
OC43/HKU1	1	0	1	
Human metapneumovirus	2	1	1	
Bocavirus	1	0	1	
Enterovirus	1	0	1	
Fungus	4 (3.1)	4 (14.8)¶	0	<0.01
*Aspergillus* species	4 (3.1)	4 (14.8)	0	
* Pneumocystis jirovecii*	1 (0.8)	1 (3.7)	0	

*Categories of co-infection were not mutually exclusive; some cases were associated with >2 categories of pathogens. †Three patients were co-infected with 2 bacteria: *H. influenzae* and *S. pneumoniae*; *E. coli* and *K. pneumoniae*; and *A. baumannii* and *K. pneumoniae*. ‡One patient was co-infected with influenza A virus and human metapneumovirus. §One patient was co-infected with bocavirus and rhinovirus. ¶One patient was co-infected with *Aspergillus* species and *P. jirovecii*.

### Clinical Manifestations and Laboratory Findings

Dyspnea, fever, sputum, and cough were the most common signs and symptoms in both groups (Table 4). Fever tended to be less common in the *C. striatum* group (66.7% vs. 82.5%; p = 0.07). The proportion of patients with septic shock at the time of ICU admission was significantly higher in the MRSA group (67.0% vs. 44.4%; p = 0.03). However, the proportion of mechanical ventilation, acute physiology and chronic health evaluation (APACHE II) score, and sequential organ failure assessment (SOFA) score at the time of ICU admission were similar between the 2 groups. Peripheral leukocyte counts, platelet counts, and serum C-reactive protein levels also were similar between the 2 groups, but serum procalcitonin level was significantly higher in the MRSA group than the *C. striatum* group (median 0.3 ng/mL vs. 1.8 ng/mL; p<0.01).

**Table 4:** Clinical and laboratory characteristics of patients with severe *Corynebacterium striatum* pneumonia and methicillin-resistant *Staphylococcus aureus* pneumonia, Seoul, South Korea, 2014–2019*

Characteristics	Total, n = 130	*C. striatum*, n = 27	MRSA, n = 103	p value
Clinical manifestation				
Dyspnea	106 (81.5)	25 (92.6)	81 (78.6)	0.16
Fever, temperature >38°C	103 (79.2)	18 (66.7)	85 (82.5)	0.07
Sputum	92 (70.8)	16 (59.3)	76 (73.8)	0.14
Cough	57 (43.8)	11 (40.7)	46 (44.7)	0.72
Altered mental status	46 (35.4)	10 (37.0)	36 (35.0)	0.84
Diarrhea	4 (3.1)	2 (7.4)	2 (1.9)	0.19
Septic shock at ICU admission	81 (62.3)	12 (44.4)	69 (67.0)	0.03
Mechanical ventilation	127 (97.7)	27 (100)	100 (97.1)	1.00
APACHE II score, mean (SD)	25.6 (8.1)	26.4 (11.9)	26.0 (7.0)	0.72
SOFA score, mean (SD)	9.5 (3.7)	9.5 (3.4)	9.5 (3.7)	0.99
Bacteremia	19 (14.6)	1 (3.7)	18 (17.5)	0.12
Laboratory findings, median (IQR)				
Leukocyte count, cells/mL	10,950 (7,800–15,625)	11,600 (4,800–15,900)	10,700 (8,400–15,600)	0.26
Platelets, × 10^3^/mL	159 (81–242)	123 (55–230)	171 (102–245)	0.14
C-reactive protein, mg/dL	11.3 (5.5–19.3)	13.6 (8.0–19.8)	10.8 (5.4–18.6)	0.61
Procalcitonin, ng/mL	1.1 (0.3–3.9)	0.3 (0.1–1.3)	1.8 (0.4–4.2)	<0.01

*Values are no. (%) except as indicated APACHE, acute physiology and chronic health evaluation; BAL, bronchoalveolar lavage; ICU, intensive care unit; IQR, interquartile range; MRSA, methicillin-resistant *Staphylococcus aureus*; SOFA, sequential organ failure assessment.

### *C. striatum* Gram Stain, Culture, and Antimicrobial Susceptibility Testing 

On microscopic examination of Gram stain specimens, gram-positive rods were identified in 69.2% (18/26) of specimens. Among 27 cases, 10 were quantitative cultures and 17 were semiquantitative cultures. Bacterial counts were >10^5^ CFU/mL in 8/10 quantitative cultures. Of the 17 semiquantitative culture specimens, 12 specimens were grade many (4+), 1 was grade moderate (3+), 1 grade few (2+), and 3 were grade rare (1+) (Appendix Table). All 27 *C. striatum* isolates underwent antimicrobial susceptibility testing. All isolates were resistant to penicillin, ceftriaxone, erythromycin, and ciprofloxacin, and susceptible to vancomycin, and all isolates were multidrug resistant.

### Outcomes

The mortality rates between the *C. striatum* and MRSA group showed no statistically significant differences: 30-day mortality (40.7% vs. 29.1%; p = 0.25), 60-day (48.1% vs. 42.7%; p = 0.61), and 90-day (59.3% vs. 50.5%; p = 0.42) (Table 5). In-hospital mortality rates were higher (70.4%) in the *C. striatum* group than in the MRSA group (52.4%), albeit without statistical significance (p = 0.09). Mortality rates were similar for *C. striatum* and MRSA in subgroups regardless of the patient’s immune status. We noted no statistically significant differences in the median length of ICU stay between the *C. striatum* and MRSA group, both 14 days (p = 0.33), nor in the length of hospital stay after ICU admission, 30 days for the *C. striatum* versus 29 days for the MRSA group (p = 0.48).

**Table 5:** Outcomes of adult patients with severe *Corynebacterium striatum* and methicillin-resistant *Staphylococcus aureus* pneumonia, Seoul, South Korea, 2014–2019*

Outcome	Total, n = 130	*C. striatum*, n = 27	MRSA, n = 103	p value
Death				
Total	n = 103	n = 27	n = 103	NA
30 days	41 (31.5)	11 (40.7)	30 (29.1)	0.25
60 days	57 (43.8)	14 (48.1)	44 (42.7)	0.61
90 days	68 (52.3)	16 (59.3)	52 (50.5)	0.42
In-hospital	73 (56.2)	19 (70.4)	54 (52.4)	0.09
Death among patient categories				
Nonimmunocompromised patients	n = 89	n = 13	n = 76	NA
30 days	21 (23.6)	5 (38.5)	16 (21.1)	0.18
60 days	31 (34.8)	5 (38.5)	26 (34.2)	0.76
90 days	40 (44.9)	7 (53.8)	33 (43.4)	0.49
In-hospital	40 (44.9)	7 (53.8)	33 (43.4)	0.49
Immunocompromised patients	n = 41	n = 14	n = 27	NA
30 days	20 (48.8)	6 (42.9)	14 (51.9)	0.59
60 days	26 (63.4)	8 (57.1)	18 (66.7)	0.55
90 days	28 (68.3)	9 (64.3)	19 (70.4)	0.73
In-hospital	33 (80.5)	12 (85.7)	21 (77.8)	0.69
Median ICU stay, d (IQR)	14.0 (8.0–26.3)	14.0 (9.0–27.0)	14.0 (8.0–26.0)	0.33
Median hospital stay after ICU admission, d (IQR)	29.5 (14.0–57.0)	30.0 (16.0–81.0)	29.0 (14.0–55.0)	0.48

*Values are no. (%) except as indicated. ICU, intensive care unit; MRSA, methicillin-resistant *Staphylococcus aureus*; NA, not applicable.

## Discussion

We investigated the proportion and characteristics of severe *C. striatum* pneumonia compared with severe MRSA pneumonia. Although the proportion of severe MRSA HAP greatly decreased during 2014–2019, the proportion of severe *C. striatum* pneumonia sharply increased and surpassed that of severe MRSA pneumonia. *C. striatum* pneumonia was more commonly associated with immunocompromise, viral co-infection, and fungal co-infection. Mortality rates between the *C. striatum* and MRSA groups were comparable.

We found that the proportion of severe MRSA pneumonia decreased while severe *C. striatum* pneumonia greatly increased and that *C. striatum* emerged as one of the most common pathogens in patients with severe HAP. Strengthened infection control measures during the study period might have contributed to the decline of severe MRSA pneumonia (*18*); however, severe *C. striatum* pneumonia demonstrated the opposite trend. Several possible explanations for this discrepancy exist. First, detection of *C. striatum* from respiratory specimens in clinical laboratories increased, possibly because experience among laboratory staff accumulated over time. Also, new reliable identification techniques, such as matrix-assisted laser desorption/ionization time-of-flight mass spectrometry, were introduced and enabled precise and rapid detection and identification of bacteria in clinical samples, which might have contributed to the increased reports of severe *C. striatum* pneumonia (*19*,*20*). Second, *C*. *striatum* can be resistant to infection control measures and can adhere to abiotic surfaces and form biofilms on various medical devices, such as feeding tubes, endotracheal tubes, and ventilators (*21*,*22*). Some reports documented *C. striatum* strains with resistance to high-level disinfectants, such as 2% glutaraldehyde and other biocides (*23*,*24*). These findings suggest that appropriate environmental infection control measures for *C. striatum* should be further investigated and implemented. Finally, hospital outbreaks also might have contributed to the seeming discrepancy. Colonized patients and contaminated inanimate objects could be reservoirs for prolonged outbreaks. However, when we chronologically analyzed the occurrence patterns according to time and place, we could not find any suggestions of notable outbreaks. Clinical observation alone creates difficulties and limitations in distinguishing outbreaks; therefore, future studies should include more detailed typing analysis of *C. striatum* isolates to identify and curb possible healthcare-associated outbreaks.

In this study, viral or fungal co-infection was more common in the *C. striatum* group, whereas other bacterial co-infection was more common in the MRSA group. This finding could represent the host factor because a greater proportion of *C. striatum* patients were in an immunocompromised state, which would make them vulnerable to opportunistic infections. Of note, fewer cases of bacterial coinfection were diagnosed in the *C. striatum* group, but the cause for this difference is uncertain. One possible explanation is that *C. striatum* might influence the behavior and fitness of other bacteria. A recent study reported that *Corynebacterium* species can reduce the toxicity of *Staphylococcus aureus* by exhibiting decreased hemolysin activity and displaying diminished fitness of in vivo coinfection (*25*). Further targeted studies on this issue are needed.

We found that serum procalcitonin level was higher in the MRSA group than in the *C. striatum* group (median 1.8 ng/mL vs. 0.3 ng/mL). Some studies suggest that serum procalcitonin can be used as a marker for bacterial infection and to differentiate bacterial from viral infection or noninfectious causes of inflammation (*26*,*27*). In 2017, a group of researchers in China reported that the median serum procalcitonin level of an *S. aureus* bacteremia group of patients was higher (1.18 ng/mL) than that of a coagulase-negative staphylococci bacteremia group (0.21–0.31 ng/mL) (*28*). We speculate that infections caused by low-virulence bacteria, such as *C. striatum* in our study, might have low levels of procalcitonin and this warrants further investigation.

Mortality rates were similarly high in both groups, but septic shock at the time of initial clinical manifestation was less common in the *C. striatum* group. Immunocompromised conditions were more common in the *C. striatum* group, which could suggest that *C. striatum* is less virulent than MRSA. Host factor might contribute to the development of severe *C. striatum*–associated pneumonia and the subsequent outcomes; however, we noted no statistically significant differences in mortality rates between the 2 groups after stratification by immunocompromised conditions. The existence of co-infection and pathogen types (e.g., other bacteria, viruses, fungi) involved might have affected mortality rates, but we were unable to effectively evaluate each effect because of the small number of patients in each subgroup.

The first limitation of our study is that we used a single-center design and our results might not be replicable in other centers or hospital systems. In addition, as we mentioned previously, we were not able to effectively evaluate the sole contribution of *C. striatum* because co-infection with other pathogens was common among the patient cohort. Finally, we included all *C. striatum* isolates from sputum, endotracheal aspirate, and bronchoalveolar lavage, but the cultures were mostly semiquantitative, and some of the *C. striatum* isolates might have been nonpathogenic colonizers. A 2020 study from the United States reported that normal respiratory flora appears to have caused one quarter of CAP cases (*29*), which supports our finding that bacteria previously considered as colonizers or normal flora can be a cause of pneumonia.

In conclusion, we found *C. striatum* was associated with severe HAP. Patients with severe *C. striatum* pneumonia showed similar clinical and laboratory features as patients with severe MRSA pneumonia, and both infections were associated with high mortality rates. Further investigations could clarify incidence, clinical characteristics, and outcomes of severe *C. striatum* pneumonia in critically ill adults and determine whether infections are due to colonization, or community- or healthcare-acquired infections. Clinicians should be aware of this emerging pathogen as a possible cause for severe pneumonia, especially among immunocompromised patients.

Appendix Additional information on severe pneumonia caused by *Corynebacterium striatum* in adults, Seoul, South Korea, 2014–2019.
