# Supplementary material for: Severe Pneumonia Caused by Corynebacterium striatum in Adults, Seoul, South Korea, 2014–2019
Source: Emerg Infect Dis. 2022 Nov;28(11):2147–54. doi: 10.3201/eid2811.220273 (PMC9622248; doi:10.3201/eid2811.220273)
Supplement: Appendix — Additional information on severe pneumonia caused by Corynebacterium striatum in adults, Seoul, South Korea, 2014–2019. [file 22-0273-Techapp-s1.pdf]

Article DOI: https://doi.org/10.3201/eid2811.220273

Severe Pneumonia Caused by *Corynebacterium striatum* in Adults, Seoul, South Korea, 2014–2019

Appendix

Appendix Table. Gram-stain results of direct smears from specimens and semiquantitative and quantitative culture results for 27 patients with severe *Corynebacterium striatum* pneumonia, Seoul, South Korea, 2014–2019

Case no.	Specimen type	Gram-stain results, direct specimen smears	Culture result	Date isolate obtained
1	Sputum (semiquantitative)	Gram positive cocci	Many (4+)	February 2014
2	Endotracheal aspirate sputum (quantitative)	Gram positive rods	2,000,000 CFU/mL	October 2014
3	Bronchial aspirate (quantitative)	No organisms seen	700,000 CFU/mL	March 2015
4	Endotracheal aspirate sputum (quantitative)	Gram positive rods	300,000 CFU/mL	June 2015
5	Sputum (semiquantitative)	Gram positive rods	Many (4+)	January 2016
6	Bronchoalveolar lavage (quantitative)	Gram positive cocci	5,000,000 CFU/mL	March 2016
7†	Endotracheal aspirate sputum (semiquantitative)	Gram positive cocci	Rare (1+)	April 2016
8	Endotracheal aspirate sputum (semiquantitative)	Gram positive rods	Moderate (3+)	April 2016
9	Endotracheal aspirate sputum (semiquantitative)	Gram positive rods	Many (4+)	August 2016
10†	Endotracheal aspirate sputum (semiquantitative)	No organisms seen	Rare (1+)	April 2017
11†	Bronchial aspirate (semiquantitative)	Gram positive cocci	Rare (1+)	April 2017
12	Endotracheal aspirate sputum (semiquantitative)	Gram positive rods	Many (4+)	June 2017
13	Bronchoalveolar lavage (quantitative)	No organisms seen	7,000 CFU/mL	May 2017
14	Endotracheal aspirate sputum (quantitative)	Gram positive rods	1,000,000 CFU/mL	July 2017
15	Sputum (semiquantitative)	Gram positive rods	Many (4+)	January 2018
16	Sputum (semiquantitative)	Not recorded	Many (4+)	March 2018
17	Endotracheal aspirate sputum (semiquantitative)	Gram positive rods	Many (4+)	March 2018
18	Endotracheal aspirate sputum (semiquantitative)	Gram positive rods	Many (4+)	March 2018
19	Bronchial aspirate (semiquantitative)	Gram positive rods	Many (4+)	March 2018
20	Bronchoalveolar lavage (quantitative)	Gram positive cocci	900,000 CFU/mL	July 2018
21	Endotracheal aspirate sputum (semiquantitative)	Gram positive rods	Many (4+)	March 2019
22	Endotracheal aspirate sputum (semiquantitative)	No organisms seen	Many (4+)	April 2019
23	Bronchoalveolar lavage (quantitative)	Gram positive rods	2,000,000 CFU/mL	April 2019
24	Endotracheal aspirate sputum (semiquantitative)	Gram positive rods	Many (4+)	May 2019
25	Endotracheal aspirate sputum (quantitative)	Gram positive rods	600,000 CFU/mL	August 2019
26	Bronchoalveolar lavage (quantitative)	Gram positive rods	50,000 CFU/mL	November 2019
27	Endotracheal aspirate sputum (semiquantitative)	Gram positive rods	Many (4+)	December 2019

*CFU, colony-forming unit.
†Although culture results were semiquantified as rare, these were diagnosed as severe *C. striatum* pneumonia cases because no other organisms were identified, and *C. striatum* was considered a pathogen in consultations with infectious disease specialists. Clinicians also used antimicrobial drugs targeting *C. striatum.*
